# From Brain to Muscle: The Role of Muscle Tissue in Neurodegenerative Disorders

**DOI:** 10.3390/biology13090719

**Published:** 2024-09-12

**Authors:** Elisa Duranti, Chiara Villa

**Affiliations:** School of Medicine and Surgery, University of Milano-Bicocca, 20900 Monza, Italy; e.duranti@campus.unimib.it

**Keywords:** muscle, neurodegenerative diseases, therapy

## Abstract

**Simple Summary:**

Neurodegenerative diseases (NDs), like amyotrophic lateral sclerosis (ALS), Alzheimer’s disease (AD), and Parkinson’s disease (PD), mainly impact the central nervous system, resulting in neuronal death and motor/cognitive impairments. However, recent studies have also shown a significant role of muscle tissue in the pathogenesis of these disorders, suggesting that pathological processes may co-exist in both the brain and muscle tissue. Understanding muscle involvement in NDs can provide novel therapeutic targets for developing more effective treatments for patients affected by NDs.

**Abstract:**

Neurodegenerative diseases (NDs), like amyotrophic lateral sclerosis (ALS), Alzheimer’s disease (AD), and Parkinson’s disease (PD), primarily affect the central nervous system, leading to progressive neuronal loss and motor and cognitive dysfunction. However, recent studies have revealed that muscle tissue also plays a significant role in these diseases. ALS is characterized by severe muscle wasting as a result of motor neuron degeneration, as well as alterations in gene expression, protein aggregation, and oxidative stress. Muscle atrophy and mitochondrial dysfunction are also observed in AD, which may exacerbate cognitive decline due to systemic metabolic dysregulation. PD patients exhibit muscle fiber atrophy, altered muscle composition, and α-synuclein aggregation within muscle cells, contributing to motor symptoms and disease progression. Systemic inflammation and impaired protein degradation pathways are common among these disorders, highlighting muscle tissue as a key player in disease progression. Understanding these muscle-related changes offers potential therapeutic avenues, such as targeting mitochondrial function, reducing inflammation, and promoting muscle regeneration with exercise and pharmacological interventions. This review emphasizes the importance of considering an integrative approach to neurodegenerative disease research, considering both central and peripheral pathological mechanisms, in order to develop more effective treatments and improve patient outcomes.

## 1. Introduction

Neurodegenerative diseases (NDs) encompass a group of progressive disorders characterized by the deterioration and death of nerve cells, leading to a decline in cognitive and/or motor functions [1,2]. Major NDs include amyotrophic lateral sclerosis (ALS), which affects motor neurons, leading to muscle weakness and progressive paralysis [3,4]; Alzheimer’s disease (AD), characterized by progressive memory loss and cognitive decline [5,6]; and Parkinson’s disease (PD), known for motor symptoms such as tremors and muscle stiffness due to the loss of dopaminergic neurons [7]. Despite their highly different pathophysiology and symptomatology, these diseases share several common features. A fundamental characteristic is neuronal degeneration, which manifests as the progressive loss of neurons in specific areas of the brain or spinal cord. This process affects a variety of neurons, such as those controlling movement (in ALS and PD) or those involved in memory and cognitive functions (in AD) [8,9]. Another common hallmark is the presence of abnormal protein aggregates within or around nerve cells. For instance, ALS in characterized by accumulations of TDP-43 and SOD-1 protein, AD by the formation of amyloid-β (Aβ) plaques and tau tangles, and PD by Lewy bodies containing α-synuclein (α-syn). These aggregates can disrupt cellular function and trigger inflammatory responses that further contribute to neuronal degeneration [3,10,11,12,13]. However, there is currently no definitive cure that can completely reverse the course of NDs, and the available therapeutic options are mostly focused on managing symptoms and slowing disease progression [14,15].

In NDs, muscle tissue also plays a crucial role, as the degeneration of motor neurons and alterations in neural signals lead to significant muscle function loss. In ALS, AD, and PD, muscles are directly or indirectly affected, contributing to the debilitating symptoms characteristic of these diseases [16,17]. In ALS, the progressive loss of motor neurons in the brain and spinal cord causes muscle weakness and atrophy, compromising patients’ ability to move and perform daily activities [4,18]. Though primarily associated with cognitive deficits, even in AD, sarcopenia, or the age-related loss of muscle mass, can occur, exacerbating immobility and increasing the risk of falls [19,20]. Also, in PD, a decrease in dopamine levels leads to muscle rigidity, tremors, and bradykinesia, which restrict mobility and balance [21,22]. Therefore, the importance of muscle tissue in NDs lies not only in its direct impact on patients’ quality of life but also in its potential as a therapeutic target. Interventions aimed at maintaining or improving muscle function, such as physical exercise, physiotherapy, and appropriate diet, can help slow symptom progression and enhance patients’ independence [23]. Moreover, pharmacological treatments that reduce oxidative stress and inflammation in muscles offer new promise for improving muscle conditions and the quality of life in individuals with NDs [4,24,25].

This review aims to explore muscle involvement in NDs, such as ALS, AD, and PD. It focuses primarily on muscle atrophy, mitochondrial dysfunction, protein aggregation, and inflammation, examining their involvement in disease progression and symptomatology. Understanding these muscle-related changes will help in the identification of novel pharmacological targets to improve muscle health and patient outcomes.

## 2. An Overview of Muscle Tissue: Structure and Function

Skeletal muscle is among the most dynamic and adaptable tissues in the human body. In humans, skeletal muscle accounts for around 40% of total body weight, contains 50–75% of the body’s proteins, and is responsible for 30–50% of the total body protein turnover. Muscle is composed of 75% water and 20% proteins, with the remaining 5% consisting of inorganic salts, minerals, lipids, and carbohydrates [26]. Muscle mass is generally regulated by the balance between protein synthesis and degradation, which is governed by factors such as nutritional intake, hormonal levels, physical activity, exercise, and the presence of injury or disease. Muscle protein types, including structural, contractile, and regulatory proteins, are of great scientific interest due to their significant role in mobility, exercise capacity, overall function, and health [27,28,29].

Skeletal muscle is essential for numerous bodily functions. Mechanically, its primary function is to convert chemical energy into mechanical energy, generating force and power while preserving posture and producing movements that allow for activity, participation in social and occupational settings, health maintenance, and functional independence [26]. Skeletal muscle contributes to basal energy metabolism, stores key substrates such as amino acids and carbohydrates, produces heat to regulate core temperature, and consumes the majority of oxygen and fuel used during physical activity and exercise [30,31]. Notably, skeletal muscle acts as a storage for amino acids required by other tissues, such as the skin, brain, and heart, for the synthesis of organ-specific proteins. Additionally, the release of amino acids from muscle helps to keep blood glucose levels stable during periods of fasting [32]. Reduced muscle mass impairs the body’s ability to respond to stress and chronic sickness, making it detrimental to disease prevention and health maintenance.

**Figure 1:**
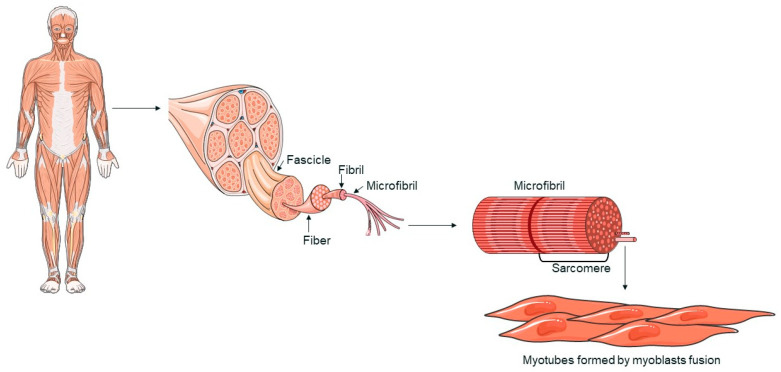
A schematic representation of skeletal muscle structure. The image was created with the use of Servier Medical Art modified templates, licensed under a Creative Common Attribution 3.0 Unported License (https://smart.servier.com, accessed on 28 August 2024).

Skeletal muscle is made up of multinucleated cells called myofibers, which are generated during development when myoblasts fuse together [33,34]. When muscle tissue is damaged, a complex response is triggered, leading to tissue regeneration [35]. This regenerative process is primarily driven by satellite cells (SCs), which respond to signals from the environment. SCs replenish myogenic progenitor cells and differentiate into new myofibers to repair muscle damage following injury [33,34]. Myofibers, the functional components of skeletal muscle, are characterized by a multinucleated structure (Figure 1). The fusion of myoblasts into myotubes, which form these fibers, is regulated by specific transcription factors, such as MyoD, Myf5, myogenin, and MRF4, collectively known as myogenic regulatory factors (MRFs) [34,36]. These factors coordinate the gene expression necessary for muscle differentiation and myofiber formation [37]. Skeletal muscle is primarily composed of myofibers, connective tissue, blood vessels, and nerves. Myofibers are organized into muscle fascicles, wrapped by the perimysium, and each individual myofiber is surrounded by the endomysium [38]. Myofibrils are cylindrical structures that extend the length of a muscle fiber. Myofibrils are composed of sarcomeres, repetitive units that represent the functional unit of muscle contraction. Sarcomeres are made up of actin and myosin filaments, whose interaction is fundamental to the muscle contraction process [39]. The regeneration of skeletal muscle is mainly mediated by SCs, stem cells located between the basal membrane and the sarcolemma of myofibers. In response to muscle damage, SCs activate and proliferate, generating myoblasts that differentiate and fuse to repair or form new myofibers [34].

## 3. The Role of Muscle Pathology in ALS: Mechanisms and Treatment Strategies

### 3.1. Overview of Pathogenic Mechanisms in ALS

ALS is a fatal ND characterized by the progressive degeneration of both upper and lower motor neurons, which contain cytoplasmic inclusions [34,40]. Upper motor neuron deterioration results in spasticity and hyperexcitability, while the lower motor neuron loss leads to weakness, fasciculations, and, ultimately, muscle atrophy, followed by progressive paralysis [3,41]. Early symptoms include muscle cramping and stiffness, progressing to muscle weakness that affects the arms and legs [34,42]. Patients often experience slurred speech and difficulties with chewing or swallowing [43]. Ultimately, death occurs as a result of respiratory failure and pneumonia complications within approximately 3–5 years after symptom onset [3]. While most ALS cases (~90–95%) are sporadic (sALS) with no known etiology, about 5–10% of cases involve familial gene mutations following a Mendelian inheritance pattern, known as familial ALS (fALS) [3,44].

The pathogenic mechanisms underlying ALS are complex, involving a combination of protein dysfunction, oxidative stress, mitochondrial dysfunction, excitotoxicity, neuroinflammation, alterations in axonal transport, and genetic factors. One of the main pathogenic processes of ALS is the formation of cytoplasmic inclusions within motor neurons. These inclusions are often composed of misfolded proteins, such as superoxide dismutase 1 (SOD1) and transactive response DNA-binding protein 43 kDa (TDP-43). The dysfunction of these proteins can cause endoplasmic reticulum (ER) stress and the activation of the unfolded protein response, ultimately leading to cell death [3,45]. Oxidative stress is another significant factor in the pathophysiology of ALS. Motor neurons are particularly vulnerable to oxidative damage due to their high metabolic activity, which results in the production of reactive oxygen species (ROS). The mutation of SOD1, an antioxidant enzyme, impairs the cells’ ability to neutralize ROS, leading to cellular damage and neuronal death [46,47]. On the other hand, mutant SOD1 can cause a global variation in structure, which may result in a gain of toxicity, exacerbating the disease [48]. Mitochondrial dysfunction plays a critical role in ALS pathogenesis. The mitochondria of affected motor neurons exhibit structural and functional abnormalities, reducing ATP generation and increasing ROS production. This energetic dysfunction promotes neuronal degeneration and disease progression [49,50]. Excitotoxicity, primarily mediated by glutamate, is another pathogenic mechanism of ALS. Affected motor neurons show a diminished capacity to remove synaptic glutamate due to the decreased expression of glutamate transporters such as EAAT2. The accumulation of extracellular glutamate causes excessive activation of N-methyl-D-aspartate (NMDA) receptors, leading to increased calcium influx into cells and triggering apoptotic pathways [51,52]. Neuroinflammation is widely recognized as a significant factor in ALS. Activated microglia and astrocytes release pro-inflammatory cytokines, such as TNF-α and IL-1β, which contribute to neuronal degeneration. Additionally, the infiltration of peripheral immune cells into the central nervous system (CNS) may exacerbate the inflammatory response, accelerating disease progression. Furthermore, accumulated protein aggregates in the brain also trigger a pro-inflammatory response through microglial activation, contributing to a neurotoxic environment. This chronic inflammatory process not only exacerbates neurodegeneration but also can further promote the accumulation of misfolded proteins, creating a vicious cycle that accelerates neuronal damage [53]. Alterations in axonal transport are also implicated in ALS pathogenesis [54]. Motor neurons rely significantly on axonal transport to distribute organelles, proteins, and other essential molecules. Mutations in genes involved in axonal transport, such as *DCTN1* and *TUBA4A,* can impair this process, leading to axonal degeneration and motor neuron death [55].

### 3.2. Involvement of Muscle Tissue in ALS Pathology

Muscle atrophy in ALS is caused primarily by the loss of neural input from degenerating motor neurons. This process begins with the degeneration of lower motor neurons, which affects neuromuscular junctions (NMJs), highly specialized synapses that ensure efficient communication between motor neurons and muscle fibers. Without these impulses, muscle fibers lose their ability to contract effectively, leading to a reduction in muscle mass and strength [56]. This process is characterized by a significant decrease in muscle fiber size, known as atrophy, and eventually results in muscle fiber loss [4,57].

Multiple cellular mechanisms contribute to muscle atrophy in ALS. One of the key pathways involved is the ubiquitin–proteasome system (UPS), which is responsible for the degradation of damaged or misfolded proteins [4]. Muscle-specific E3 ubiquitin ligases, such as MuRF1 and atrogin-1/MAFbx, are upregulated in ALS, targeting muscle proteins for degradation [58]. This increased proteolytic activity accelerates muscle wasting [56,59]. The UPS is a tightly regulated system that labels defective proteins with ubiquitin molecules, marking them for destruction by the proteasome, a large protease complex. In ALS, the dysregulation of this system leads to excessive protein degradation, contributing to the rapid loss of muscle mass [4]. Additionally, the autophagy–lysosome pathway (ALP), another crucial protein degradation system, is dysregulated in ALS. Autophagy, the process by which cells degrade and recycle their components, becomes overactive in ALS-affected muscles [60]. This results in the excessive breakdown of muscle proteins and organelles, further contributing to muscle atrophy [61,62]. The activation of autophagy is a complex phenomenon, and its role as a cause or effect of the disease remains a topic of ongoing debate [63]. This activation could be a protective response aimed at clearing toxic protein aggregates and damaged cellular components that accumulate due to ALS-related effects. However, some evidence suggests that dysregulated or excessive autophagy may contribute to neuronal damage, potentially exacerbating the disease [64,65]. Given these dual perspectives, it is crucial to further investigate whether autophagy in ALS primarily serves as a compensatory mechanism or whether it plays a more direct role in disease progression. Apoptosis, or programmed cell death, is also a significant factor in ALS muscle pathology [66,67]. The balance between pro-apoptotic and anti-apoptotic factors is disrupted in ALS, leading to increased muscle cell death. Elevated amounts of pro-apoptotic proteins such as Bax, along with decreased levels of anti-apoptotic proteins like Bcl-2, promote muscle fiber apoptosis [56,68]. This apoptotic cascade is triggered by a variety of stress signals, including mitochondrial dysfunction and oxidative stress, leading to the systematic dismantling and removal of muscle cells [56,69].

The process of denervation and reinnervation is a dynamic feature of ALS muscle pathology. Early in the disease, surviving motor neurons attempt to compensate for lost connections by sprouting new axons that reinnervate denervated muscle fibers. This compensatory reinnervation can temporarily maintain muscle function [18,70]. However, as the disease advances, the potential for reinnervation diminishes, resulting in extensive and irreversible muscle atrophy [70]. The initial phase of reinnervation involves the reorganization of the motor unit, but the relentless progression of motor neuron death eventually overwhelms the compensatory mechanisms, leading to the progressive loss of muscle function [57]. A notable aspect of ALS muscle pathology is the shift in muscle fiber types. ALS predominantly affects type II (fast-twitch) muscle fibers, which are more prone to denervation [67,71,72]. This initially preserves type I (slow-twitch) fibers, but they progressively undergo atrophy [71,72]. The shift alters the functional properties of muscles, reducing their overall strength and endurance. Fast-twitch fibers are critical for rapid, powerful movements, whereas slow-twitch fibers are essential for endurance and sustained activities. The selective vulnerability of fast-twitch fibers in ALS disrupts the balance and coordination of muscle activity, contributing to the characteristic weakness and fatigue observed in patients [73,74]. While the precise mechanisms of this selective vulnerability are not fully understood, several hypotheses have been proposed. One key factor is oxidative stress. Fast-twitch muscle fibers, which rely more heavily on anaerobic glycolysis for energy, are more prone to the accumulation of ROS. This oxidative stress can cause greater damage to these fibers compared to slow-twitch fibers, which are better equipped to handle oxidative stress due to their reliance on oxidative phosphorylation. Further support for this is provided by several studies showing that oxidative damage is particularly detrimental to fast-twitch fibers in ALS [56,75,76]. Another contributing factor is the susceptibility of fast-twitch fibers to apoptosis. These fibers seem to be more sensitive to apoptotic signals, making them more vulnerable to degeneration in ALS [72]. Additionally, the interaction between motor neurons and muscle fibers plays a role: fast-twitch fibers are often innervated by motor neurons that are more susceptible to degeneration in ALS. As these motor neurons deteriorate, the fast-twitch fibers they control are more likely to be affected [77].

**Figure 2:**
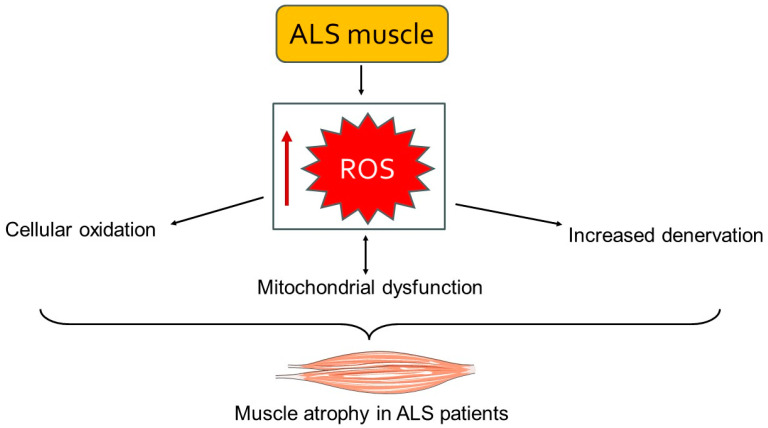
A schematic representation of the accumulation of ROS leading to mitochondrial dysfunction and cellular damage. This cascade of events results in muscle atrophy and weakness. The image was created with the use of Servier Medical Art modified templates, licensed under a Creative Common Attribution 3.0 Unported License (https://smart.servier.com, accessed on 28 August 2024).

Mitochondrial dysfunction is another important element of muscle involvement in ALS [4]. Mitochondria in ALS-affected muscles exhibit structural abnormalities, such as swollen and fragmented cristae, which impair their function. These mitochondrial defects reduce ATP synthesis while increasing ROS production [78]. The resulting bioenergetic deficits and oxidative stress further damage muscle cells and contribute to the progression of muscle atrophy [79] (Figure 2). Mitochondria are the powerhouses of the cell, and their dysfunction in ALS leads to an energy crisis within muscle cells, compromising their viability and function. Moreover, the accumulation of ROS causes oxidative damage to proteins, lipids, and DNA, further exacerbating muscle cell death [4,56]. Inflammation and oxidative stress are significant contributors to ALS muscle damage [80,81]. Pro-inflammatory cytokines such as TNF-α, IL-1β, and IL-6 are elevated in ALS muscle tissue. These cytokines activate signaling pathways that exacerbate muscle atrophy and fibrosis [80]. Additionally, oxidative stress markers, including lipid peroxidation and protein carbonylation, are increased in ALS muscles, indicating greater oxidative damage. Chronic inflammation and oxidative stress generate a hostile environment within the muscle tissue, promoting catabolic processes while inhibiting regenerative efforts [56,82].

In ALS, the integrity of the NMJ is compromised, limiting synaptic transmission and contributing to muscle weakness [83]. Structural changes in the NMJ include the fragmentation of postsynaptic acetylcholine receptor clusters and a reduction in presynaptic vesicles [4]. These alterations hinder the effective transmission of nerve signals to muscles, exacerbating muscle atrophy and weakness [84]. In ALS, the disruption of NMJ architecture affects neuromuscular transmission, further contributing to muscle function loss [85,86].

Muscle regeneration in ALS is severely impaired due to malfunctioning SCs, the resident stem cells responsible for muscle maintenance and repair [57,87]. In ALS, their number is reduced, and their ability to proliferate and differentiate is compromised [4,67]. This is partly due to the decreased expression of MRFs, such as MyoD and myogenin, which are essential for muscle regeneration [36]. As a result, the muscle’s ability to repair itself in response to damage is considerably reduced [4,57].

### 3.3. Therapeutic Strategies for Muscle Tissue in ALS

#### 3.3.1. Pharmacological Treatments

Therapeutic options for ALS aim to delay disease progression, manage symptoms, and improve the quality of life for patients [42]. In the past decade, only four drugs have been approved by the FDA for ALS treatment, with differing regulatory approvals worldwide: riluzole, edaravone, the combination therapy AMX0035, and tofersen [4,88].

Since 1995, the only medication approved for ALS treatment is riluzole, which inhibits glutamate release. Riluzole can halt disease progression and extend the median survival time by approximately three to six months, although it might cause side effects such as liver problems and diarrhea [89,90]. Despite its benefits, riluzole has not been shown to improve motor neuron function, lung function, fasciculations, or muscle strength. Its mechanism of action involves inhibiting voltage-gated sodium channels in the CNS, thereby reducing calcium influx and glutamate-induced toxicity in the motor cortex and spinal cord. Additionally, riluzole may have antioxidant properties, which aid in alleviating oxidative stress generated by various oxidizing agents [91,92]. Research on this drug in the muscular field is still in its early stages, and it appears that its effects on muscles are indirect and limited.

Radicava (edaravone) was approved by the FDA in 2017 for the treatment of ALS patients, recognizing its potential therapeutic benefits, as demonstrated in several trials. Edaravone acts as a free radical scavenger, reducing lipid peroxides in a manner comparable to antioxidants like vitamin E and ascorbic acid [93,94,95]. It targets hydroxyl radicals, peroxynitrites, and other ROS [96,97]. This drug protects neuronal, glial, endothelial vascular, and muscle cells against oxidative stress [4]. However, the precise mechanism by which edaravone exerts its effects in ALS patients remains unclear, and its direct benefits for muscle tissue are still being investigated.

In September 2022, the FDA also approved AMX0035, which is believed to mitigate neuronal cell death by reducing ER stress and mitochondrial dysfunction [98]. Specifically, this drug operates through a dual mechanism to address neurodegeneration, combining two active compounds: sodium phenylbutyrate and taurursodiol. Sodium phenylbutyrate acts as a chemical chaperone, which alleviates ER stress, whereas taurursodiol, a bile acid, supports mitochondrial function and mitigates oxidative stress. By targeting these cellular stress responses, AMX0035 reduces the levels of oxidative stress and inhibits apoptosis in motor neurons, delaying the course of ALS [99,100]. This multifaceted approach aims to protect motor neurons from damage and preserve their function, offering a significant advancement in the treatment of this debilitating disease [97]. In a later open-label extension study, patients treated with AMX0035 exhibited a significantly slower decline in physical function and a longer median overall survival compared to those given a placebo during the phase 2 CENTAUR clinical trial. Clinical trials have shown that AMX0035 can reduce the loss of physical function in ALS patients, suggesting that it might help retain muscle mass and strength [4,100,101,102].

Last year, in April, tofersen received FDA approval based on the results of phase 3 VALOR clinical trial results for ALS patients with a mutation in the *SOD1* gene [103]. Tofersen is an antisense oligonucleotide designed to lower SOD1 concentrations in the cerebrospinal fluid (CSF) and reduce plasmatic levels of neurofilament light chain (NfL), a marker of neurodegeneration [4]. Although tofersen did not improve clinical endpoints and was associated with adverse effects, a phase 3 open-label extension study (ATLAS) is ongoing to evaluate its clinical benefits in patients with presymptomatic SOD1-ALS. Although its direct therapeutic effects on muscles are still being investigated, tofersen has shown the ability to reduce the levels of NfL, which could have a favorable impact on muscle health [4,103,104,105].

#### 3.3.2. Therapeutic Potential of Exercise for Muscle Preservation in ALS

Regular physical activity has considerable benefits for patients with neuromuscular disease, including ALS, since it may slow muscle degeneration and preserve NMJ integrity [106]. Exercise activates muscle metabolism, enhances glucose utilization, and supports muscle regeneration. It also improves antioxidant capacity, mitochondrial biogenesis, and neurogenesis [107,108]. Research explores these benefits and investigates therapeutic applications cautiously, as incorrect application can lead to adverse effects. Moderate-intensity training mitigates cell damage from inflammation and retains muscle mitochondrial function with aging, contrasting with high-intensity training effects [4,109,110]. In ALS models, moderate exercise improves the phenotypes of SOD1-G93A mice, with aquatic activities like swimming extending the lifespan more effectively than running. Studies suggest swimming’s impact on motor units may explain this discrepancy [111,112,113]. Exercising, particularly swimming, in ALS models has been linked to the dysregulation of the BDNF/TrkB pathway due to muscle-contraction-induced BDNF over-secretion, which may exacerbate neurodegeneration [114,115]. Human studies indicate that exercise therapy improves ALS patients’ physical conditions and quality of life compared to standard therapy alone, highlighting its therapeutic promise, pending more targeted studies for optimized treatment options [116,117].

## 4. Exploring Muscle Pathology in Alzheimer’s Disease: Current Research and Future Directions

### 4.1. Overview of Pathogenic Mechanisms in AD

AD is an age-related ND and the most common form of dementia among the elderly population globally, accounting for up to 80% of all diagnoses [118]. AD is clinically defined as irreversible and progressive neurodegeneration characterized by initial memory loss and cognitive impairments that influence speech, behavior, motor system, and visuospatial orientation, ultimately resulting in an autonomy loss that requires full-time medical care [119]. Brain atrophy, the extracellular deposition of senile plaques made of insoluble Aβ peptide, and the intracellular formation of neurofibrillary tangles (NFTs) formed by hyperphosphorylated twisted filaments of the microtubule-associated protein tau are the major pathological hallmarks of AD [120,121]. AD pathogenesis involves not only Aβ and tau pathology but also microglia-mediated inflammation, oxidative stress, mitochondrial dysfunction, and synaptic damage. These pathways may be linked to cognitive decline, indicating a complex etiology [122].

The majority of AD cases are sporadic with a late onset (LOAD), and they frequently affect people aged 65 or older. The two primary risk factors for AD are aging and carrying the ε4 allele of the *APOE* gene, which encodes Apolipoprotein E (ApoE) [123]. On the other hand, the uncommon early-onset forms of AD (EOAD) usually affect people under the age of 65 and have an autosomal dominant inheritance pattern. These forms are caused by mutations in presenilin-1, presenilin-2, and amyloid precursor protein (APP), which are encoded by the genes *PSEN1*, *PSEN2*, and *APP*, respectively [124]. They all contribute to APP maturation and processing, resulting in an increase in Aβ synthesis or aggregation [125].

### 4.2. The Impact of Muscle Tissue on AD Pathology

The role of muscle tissue in AD is a developing area of research that could provide new insights into the disease mechanisms and potential therapeutic interventions [126]. While AD research has historically focused on the brain, it is becoming increasingly clear that the disease has systemic implications, including significant effects on muscle tissue [127].

Alterations in muscle tissue in patients with AD are multifactorial and can be attributed to various pathophysiological mechanisms [127,128]. One of the key mechanisms is mitochondrial dysfunction, which has been extensively documented in the brains of AD patients [129]. This mitochondrial dysfunction is not limited to the brain but also affects skeletal muscles, leading to reduced ATP production and increased oxidative stress. Muscle cells with malfunctioning mitochondria are unable to maintain efficient energy metabolism, resulting in muscle weakness and fatigue. The inability of mitochondria to generate appropriate energy disrupts normal muscle function and contributes to the overall decline in physical capabilities observed in AD patients [130,131,132,133]. Additionally, the accumulation of Aβ has also been observed in skeletal muscles. This toxic peptide can interfere with muscle function through various mechanisms, including the induction of oxidative stress, ER dysfunction, and the activation of pro-apoptotic pathways [134,135]. The accumulation of Aβ in muscles may directly contribute to the muscle mass loss and muscle dysfunction observed in AD patients. Therapies targeting Aβ may benefit both neurological and muscle systems due to their pathological overlap [134,136,137].

Another critical factor is neuroinflammation, which plays a central role in the pathogenesis of AD. Neuroinflammation is characterized by the chronic activation of glial cells and the release of pro-inflammatory cytokines [138], which can spread systemically and negatively impact muscle tissue, inducing a local inflammatory state [139]. Chronic inflammation in muscles can lead to protein degradation and reduced protein synthesis, further contributing to the sarcopenia associated with AD. The systemic nature of inflammation highlights the importance of addressing inflammatory pathways in the treatment of AD [140,141].

Recent studies have also highlighted alterations in insulin signaling in AD patients, which can affect muscle metabolism. Insulin resistance, common in AD patients, can impair glucose uptake in muscles and alter muscle energy metabolism [142,143,144]. This can cause lower protein synthesis and increased protein degradation in muscles, exacerbating muscle mass loss. Insulin resistance not only affects brain glucose metabolism but also has a significant impact on peripheral tissues, implying that metabolic interventions could be beneficial [145,146].

The loss of muscle mass and function in AD patients not only compromises mobility and independence but also can worsen cognitive decline [127,147,148,149]. Reduced physical activity due to muscle weakness can contribute to further cognitive deterioration through various mechanisms, including reduced cerebral blood flow and increased systemic inflammation. This bidirectional relationship between muscle and cognitive function suggests that preserving muscle health could have significant benefits for overall disease management [147,148].

### 4.3. Therapeutic Approaches for Muscle Tissue in AD

#### 4.3.1. Pharmacological Treatments

Pharmacological interventions targeting systemic inflammation may mitigate muscle damage in AD patients. Nonsteroidal anti-inflammatory drugs (NSAIDs) and other anti-inflammatory agents can potentially reduce inflammatory cytokine levels and muscle inflammation [150,151]. Additionally, agents that enhance mitochondrial function, such as coenzyme Q10 and creatine, can help preserve muscle energy metabolism and prevent atrophy [152,153].

Neuromuscular electrical stimulation (NMES) is the process of causing muscles to contract by sending electrical impulses to them. This therapy can help maintain muscle mass and strength in AD patients with limited mobility [154]. NMES has shown promise in improving muscle function and physical performance in older adults, particularly those affected by NDs. By stimulating muscle contraction and enhancing blood flow, NMES can counteract the effects of reduced physical activity in AD patients.

Hormones such as growth hormone and insulin-like growth factor-1 (IGF-1) play crucial roles in muscle growth and repair [155]. Hormone supplementation has the potential to reverse muscle atrophy in AD patients. However, the safety and efficacy of such treatments require further investigation. Studies on the use of these hormones in other muscle-wasting conditions suggest potential benefits, but their specific effects on AD need to be explored in greater detail [155,156]. Ongoing research is focused on understanding the molecular mechanisms linking AD pathology to muscle degeneration. Identifying new therapeutic targets within these pathways could lead to innovative treatments [157,158,159]. Combining multiple therapeutic approaches, including exercise, nutritional support, pharmacological treatments, and NMES, may offer synergistic benefits. Personalized medicine approaches that tailor interventions to individual patient profiles hold promise for improving outcomes.

#### 4.3.2. Therapeutic Potential of Exercise for Muscle Preservation in AD

Muscle tissue in AD patients undergoes several pathological changes, including muscle fiber atrophy, increased presence of type II fibers, mitochondrial dysfunction, and heightened oxidative stress [160,161]. The degeneration of the central nervous system (CNS) disrupts neuromuscular signaling, leading to reduced muscle use and subsequent atrophy. Additionally, systemic inflammation and metabolic dysfunction associated with AD exacerbate muscle degeneration.

Regular physical exercise is one of the most effective interventions for improving muscle health in AD patients. Exercise enhances muscle strength, improves mitochondrial function, and reduces oxidative stress [162,163]. Aerobic exercises, such as walking and cycling, improve cardiovascular health and muscle endurance, while resistance training increases muscle mass and strength [164]. Exercise also promotes the release of neurotrophic factors like brain-derived neurotrophic factor (BDNF), which supports neuronal health and function [165]. Tailoring exercise programs to the capabilities and limitations of AD patients is essential for maximizing benefits and minimizing risks [166].

In addition to physical exercise, this condition requires a proper nutritional program [167]. Adequate nutrition is crucial for maintaining and repairing muscle tissue [168]. Protein supplementation is vital for counteracting muscle atrophy, providing the necessary building blocks for muscle repair and growth [169]. Antioxidants, such as vitamins E and C and omega-3 fatty acids, help reduce oxidative stress and inflammation, supporting overall muscle health [170,171]. Ensuring that AD patients receive a balanced and adequate diet can significantly impact their muscle function and quality of life [172].

## 5. Muscle Dysfunctions in PD: Implications for Motor Symptoms and Therapeutic Strategies

### 5.1. Overview of Pathogenic Mechanisms in PD

PD is a progressive ND primarily characterized by the degeneration of dopaminergic neurons in the substantia nigra pars compacta, a region in the midbrain [173,174]. This process leads to decreased dopamine levels in the striatum, adversely affecting motor control and resulting in the characteristic symptoms of the disease, such as resting tremor, muscle rigidity, bradykinesia, and postural instability [22,175].

The pathogenesis of PD is complex and multifactorial, arising from the interplay of genetic and environmental factors. Approximately 10–15% of PD cases are familial, indicating a strong genetic component [176,177]. Several genes have been associated with the disease, including *SNCA* (α-syn), *LRRK2* (leucine-rich repeat kinase 2), *PARK2* (parkin), *PARK7* (DJ-1), and *PINK1* (PTEN-induced kinase 1). Mutations in these genes can lead to cellular dysfunctions that contribute to neurodegeneration [178,179]. A key pathological hallmark of PD is the presence of Lewy bodies, eosinophilic cytoplasmic inclusions primarily composed of aggregated α-syn [180,181]. Alpha-syn plays a crucial role in the pathogenesis of PD. Under normal conditions, α-syn regulates synaptic function and neuronal plasticity. However, in pathological conditions, the protein can misfold and aggregate, forming toxic oligomers and insoluble fibrils that contribute to synaptic dysfunction and neuronal death [182,183].

Mitochondrial dysfunction and oxidative stress are also pivotal in PD pathogenesis [184,185]. Mitochondrial dysfunction leads to the excessive production of ROS, resulting in oxidative stress, damage to cellular macromolecules, and neuronal death. Mutations in the *PINK1* and *PARK2* genes, which are involved in mitophagy, the selective removal of damaged mitochondria, can impair mitochondrial function, increasing susceptibility to neurodegeneration [186,187,188,189].

Autophagy, a cellular process essential for degrading and recycling damaged proteins and organelles, is also disrupted in PD [190,191]. This impairment leads to the accumulation of misfolded proteins and cellular debris. UPS dysfunction, another crucial pathway for protein degradation, has also been implicated in PD pathogenesis [192,193]. Dysfunctions in these proteostasis pathways can result in the accumulation of α-syn and other toxic proteins.

Neuroinflammation contributes significantly to the progression of PD [194,195]. When neurons degenerate, microglial cells, the resident macrophages of the CNS, become activated and release pro-inflammatory cytokines, ROS, and other inflammatory molecules. This inflammatory environment can further damage neurons and contribute to disease progression. Chronic microglial activation and systemic inflammation have been correlated with the severity of PD [196,197].

Various cellular signaling pathways are altered in PD. The LRRK2 kinase pathway is of particular interest, as mutations in *LRRK2* are among the most common genetic causes of PD [198]. LRRK2 is involved in various cellular processes, including vesicular trafficking, autophagy, and immune signaling. Mutations in *LRRK2* can lead to its hyperactivation, causing multiple cellular dysfunctions [199,200].

### 5.2. The Role of Muscle Tissue on PD Pathology

The importance of peripheral tissues, particularly muscle tissue, in PD pathophysiology has gained increasing attention. Recent evidence suggests that muscle tissue not only undergoes secondary changes due to neural degeneration but also may actively contribute to disease progression and symptomatology [201]. Muscle tissue in PD patients reveals various structural and functional alterations [202]. These changes are primarily attributed to reduced neural input resulting from dopaminergic neuron degeneration and the consequent motor deficits. Histopathological analysis of muscle biopsies from PD patients has revealed several characteristic changes, including muscle fiber atrophy, type II fiber predominance, and the increased presence of mitochondrial abnormalities [203]. Muscle fiber atrophy, particularly in type II (fast-twitch) fibers, is commonly observed. This atrophy is likely a consequence of denervation and disuse, secondary to the impaired motor function in PD. There is also a shift toward a higher proportion of type I (slow-twitch) fibers [204]. This alteration in fiber-type composition may be an adaptive response to the chronic nature of PD, favoring endurance over rapid, forceful contractions. Muscle biopsies frequently reveal an increased presence of abnormal mitochondria, including swelling, disrupted cristae, and the accumulation of electron-dense material. Mitochondrial dysfunction plays a critical role in PD pathogenesis, affecting both the CNS and peripheral tissues such as muscle [205,206]. Key features of mitochondrial dysfunction in PD muscle tissue include reduced respiratory chain activity, oxidative stress, and genetic mutations [207]. Muscle biopsies from PD patients exhibit decreased activity of mitochondrial respiratory chain complex I, which is consistent with studies of the substantia nigra [208,209]. This reduction in complex I activity impairs oxidative phosphorylation and ATP synthesis. Mitochondrial dysfunction is also associated with the increased production of ROS, leading to oxidative damage to cellular components, including lipids, proteins, and DNA [210,211]. Oxidative stress further exacerbates mitochondrial impairment and muscle damage. Additionally, mutations in genes such as *PINK1* and *PARK2*, which are involved in mitochondrial quality control through mitophagy, have been implicated in PD. Defects in these pathways lead to the accumulation of dysfunctional mitochondria in muscle cells, contributing to muscle degeneration [212,213].

**Figure 3:**
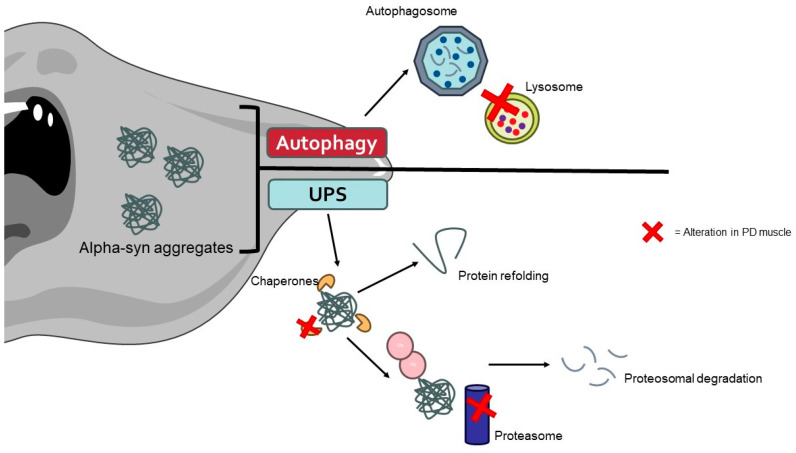
The aggregates of α-synuclein caused by alterations in protein degradation pathways promote the muscle cell alterations typically found in PD patients. The image was created with the use of Servier Medical Art modified templates, licensed under a Creative Common Attribution 3.0 Unported License (https://smart.servier.com, accessed on 28 August 2024).

Recent studies have identified the presence of α-syn aggregates in the muscle tissues of PD patients [214]. These aggregates can disrupt cellular homeostasis and contribute to muscle pathology through impaired protein degradation, cytotoxicity, and neuronal–muscular crosstalk. Alpha-syn aggregates can inhibit the UPS system and autophagy, leading to the accumulation of damaged proteins and organelles in muscle cells (Figure 3) [215,216]. In the UPS, α-syn aggregates can physically obstruct the proteasome’s active sites, preventing the degradation of ubiquitinated proteins. Additionally, these aggregates can impair the function of the proteasome by interacting with its subunits, resulting in a decrease in proteasomal activity. This leads to the accumulation of undegraded and damaged proteins, which contributes to cellular stress and dysfunction [217].

The presence of α-syn aggregates can also induce cytotoxic effects, including the disruption of cellular membranes, interference with synaptic function, and the triggering of apoptosis [218]. Alpha-syn pathology in muscle tissue may reflect the spread of pathogenic protein species from the CNS to peripheral tissues, highlighting a bidirectional relationship between neuronal and muscular degeneration in PD [219,220].

Neuroinflammation is a key feature of PD, and there is growing evidence that peripheral inflammation, including within muscle tissue, may contribute to disease progression [221,222]. Elevated levels of pro-inflammatory cytokines, such as TNF-α, IL-1β, and IL-6, have been detected in the sural nerves of PD patients [223]. These cytokines can exacerbate muscle degeneration and contribute to the overall disease burden. Immune cells, including macrophages and T lymphocytes, have been found to infiltrate PD muscle tissue more frequently [223,224]. This immune response may be triggered by the release of damage-associated molecular patterns (DAMPs) from degenerating muscle cells [225,226]. Chronic systemic inflammation, as observed in PD, can have deleterious effects on muscle tissue, promoting catabolic pathways and muscle wasting [227].

### 5.3. Therapeutic Interventions for Muscle Tissue in PD

#### 5.3.1. Pharmacological Treatments

Pharmacological interventions targeting mitochondrial dysfunction and oxidative stress are also being investigated. Mitochondrial enhancers such as coenzyme Q10 and creatine have shown potential in improving mitochondrial function [228]. Coenzyme Q10 is a component of the electron transport chain and is essential for mitochondrial ATP production. While early studies suggested potential benefits, larger clinical trials revealed conflicting results. Creatine, another mitochondrial enhancer, has shown promise in preclinical models but requires further investigation in clinical trials [229,230,231].

Antioxidants such as N-acetylcysteine (NAC) and alpha-lipoic acid (ALA) aim to mitigate oxidative stress in muscle cells. NAC acts as a precursor to glutathione, a major cellular antioxidant, while ALA is a cofactor for mitochondrial enzymes and a potent antioxidant [232]. These compounds have shown potential in reducing oxidative damage and preserving muscle function, but larger clinical trials are needed to confirm their efficacy in PD [233].

Chronic inflammation contributes to muscle pathology in PD, and anti-inflammatory drugs such as NSAIDs and corticosteroids may help mitigate this [234]. NSAIDs inhibit cyclooxygenase (COX) enzymes, reducing the production of pro-inflammatory prostaglandins [235]. Corticosteroids, on the other hand, suppress a broad range of inflammatory pathways. These agents could reduce muscle inflammation and degeneration, although their long-term use must be carefully managed due to potential side effects.

Adequate protein intake is essential for muscle repair and growth. Protein supplements, such as whey protein, provide a convenient way to ensure that PD patients receive sufficient protein, especially those with a reduced appetite or difficulty eating. Whey protein contains important amino acids, particularly leucine, which is critical for muscle protein synthesis. Branched-chain amino acids (BCAAs), including leucine, isoleucine, and valine, are particularly effective in stimulating muscle protein synthesis [236,237,238,239]. Leucine, in particular, activates the mTOR pathway, which is crucial for muscle growth and repair [240]. Supplementing with BCAAs may help prevent muscle wasting and improve muscle strength in PD patients [241,242].

#### 5.3.2. Therapeutic Potential of Exercise for Muscle Preservation in PD

Exercise has been shown to have neuroprotective effects in PD, potentially mediated through its impact on muscle tissue. Regular physical activity can improve muscle strength, enhance mitochondrial function, and reduce inflammation. Exercise-induced muscle contraction stimulates the production of neurotrophic factors such as BDNF, which can cross the blood–brain barrier and exert protective effects on dopaminergic neurons [243]. Additionally, exercise promotes autophagy, which aids in the clearance of damaged proteins and organelles in muscle cells, potentially reducing the pathological burden in both muscle and neural tissues.

Aerobic exercise is particularly beneficial for cardiovascular health and muscle endurance in PD patients. It enhances mitochondrial biogenesis, increasing the number and function of mitochondria within muscle cells, which is critical given the mitochondrial dysfunction observed in PD [244,245]. Aerobic activities, such as brisk walking, cycling, and swimming, also promote neurogenesis and the release of neurotrophic factors like BDNF, which support neuronal health and synaptic plasticity. Studies have demonstrated that regular aerobic exercise can improve motor function, reduce bradykinesia, and enhance the overall quality of life in PD patients [246,247].

Resistance training, which involves lifting weights or using resistance bands, can significantly increase muscle mass and strength. This type of exercise stimulates muscle protein synthesis and counteracts the sarcopenia (muscle wasting) commonly observed in PD patients [248]. Research shows that resistance training can improve muscle strength, enhance balance and coordination, and reduce the risk of falls, which are common in PD due to postural instability [249,250].

Flexibility and balance exercises, such as yoga and tai chi, are critical for preserving joint range of motion and enhancing proprioception. These exercises improve flexibility, balance, and coordination, reducing the risk of falls and improving functional mobility. Tai chi, in particular, has been shown to improve balance and reduce the number of falls in PD patients through its slow, controlled movements and emphasis on body awareness [251,252,253].

## 6. Muscle Degeneration across Neurodegenerative Disorders: Comparative Insights from ALS, AD, and PD

### 6.1. Similarities in the Effects on Muscle Tissue

#### 6.1.1. Muscle Atrophy and Weakness

In all three NDs, weakness and muscle atrophy are common pathological features [254]. Muscle atrophy occurs due to a combination of factors, including denervation, reduced motor neuron input, and the systemic effects of chronic disease [255]. In ALS and PD, the progressive loss of motor neurons directly affects the muscle tissue, leading to atrophy and weakness [256]. AD patients also exhibit muscle wasting, which may be due to generalized neurodegeneration and the physical inactivity associated with cognitive decline [257].

#### 6.1.2. Mitochondrial Dysfunction

Mitochondrial dysfunction characterizes the pathophysiology of all three NDs, affecting both neural and muscle tissues [258]. ALS muscle pathology also involves mitochondrial dysfunction, which is caused by defects in mitochondrial dynamics and bioenergetics that are linked to muscle weakness and atrophy [4]. Mitochondrial abnormalities are observed in the muscle tissues of AD patients, where reduced mitochondrial function contributes to decreased energy production and increased oxidative stress [259]. In PD, similar mitochondrial dysfunction is evident in both the substantia nigra neurons and muscle cells, characterized by impaired mitochondrial respiratory chain complex I activity [205,260]. Across these diseases, mitochondrial dysfunction leads to reduced ATP production, increased ROS, and subsequent muscle fatigue and degeneration.

#### 6.1.3. Protein Aggregation

Abnormal protein aggregation is a critical pathogenic feature in ALS, AD, and PD, affecting both the CNS and peripheral tissues such as muscle [261]. In ALS, the aggregation of proteins such as TDP-43 and SOD1 within muscle cells parallels their aggregation in motor neurons, leading to muscle pathology and dysfunction [3,4]. These protein aggregates interfere with normal cellular functions, including proteostasis, autophagy, and intracellular transport, further exacerbating muscle degeneration. In AD, Aβ and tau protein aggregates are found in both the brain and muscle tissues, contributing to cytotoxicity and muscle degeneration [262]. Similarly, in PD, α-syn aggregates form Lewy bodies in the brain and have been detected in muscle tissue, compromising cellular homeostasis [214].

#### 6.1.4. Inflammatory Process

Neuroinflammation plays a significant role in the progression of ALS, AD, and PD and is also evident in the muscle tissues of affected patients [222]. ALS pathology involves widespread inflammation, with inflammatory markers and immune cells detected in muscle biopsies from patients [4]. Inflammation exacerbates muscle damage through mechanisms such as increased oxidative stress, the disruption of muscle repair processes, and the release of pro-inflammatory mediators. AD is characterized by chronic inflammation in the brain, with systemic inflammation potentially impacting muscle health and function [263]. In PD, elevated levels of pro-inflammatory cytokines and immune cell infiltration are observed in both the brain and muscle tissues, contributing to muscle degeneration [264,265].

### 6.2. Specific Differences between the Diseases

#### 6.2.1. Mechanisms of Motor Neuron Degeneration

The primary pathological mechanisms leading to muscle involvement differ significantly among ALS, AD, and PD [266]. ALS is directly characterized by the degeneration of both upper and lower motor neurons, leading to rapid and severe muscle atrophy and weakness as a primary feature of the disease [3]. AD is primarily a cognitive disorder, with muscle pathology developing as a result of overall physical decline and reduced activity rather than direct motor neuron degeneration [267]. In contrast, the motor symptoms of PD, including bradykinesia and rigidity, result in decreased physical activity and secondary muscle wasting [268].

#### 6.2.2. Disease Onset and Progression

The onset and progression of muscle pathology vary between ALS, AD, and PD. In ALS, muscle pathology is rapid and severe from the onset, with patients experiencing significant muscle weakness and atrophy early in the disease course [4,57]. The rapid progression in ALS contrasts sharply with the more gradual muscle involvement seen in AD and PD. AD typically begins with cognitive decline, with muscle involvement occurring later in the disease course as physical function deteriorates [269,270]. In PD, muscle involvement is gradual, correlating with the slow progression of motor symptoms. Patients often maintain some amount of muscle function for many years following diagnosis.

#### 6.2.3. Therapeutic Responses

Therapeutic strategies targeting muscle pathology also differ among these diseases. ALS therapies focus on slowing disease progression and managing symptoms, with limited options for directly improving muscle health. Exercise may help maintain muscle function for a time, but the rapid progression of muscle atrophy in ALS limits the long-term benefits [271]. In AD, physical activity is recommended to improve general health and cognitive function, but specific interventions targeting muscle pathology are less developed [272]. In PD, exercise and physical therapy are widely recognized for their benefits in improving muscle strength, flexibility, and overall motor function. Aerobic and resistance training are particularly effective in mitigating muscle atrophy and improving the quality of life [244,245].

#### 6.2.4. Molecular Pathways

The molecular pathways involved in muscle pathology differ significantly among these disorders. ALS muscle pathology is closely linked to disruptions in RNA processing and protein homeostasis, with mutations in genes such as *SOD1*, *TARDBP*, and *FUS* playing critical roles [3,273]. AD muscle pathology involves pathways related to APP metabolism and tau phosphorylation, with a lower emphasis on mitochondrial dysfunction than in PD [274]. Muscle degeneration in PD is characterized by mitochondrial dysfunction and oxidative stress, with specific pathways such as PINK1/Parkin-mediated mitophagy being implicated [212,213]. These molecular differences underscore the distinct pathological mechanisms driving muscle involvement in each disease.

#### 6.2.5. Role of Different Quality Control Mechanisms

In the context of NDs, both autophagy and the UPS play crucial roles in maintaining cellular homeostasis by managing protein degradation and organelle turnover. However, their functions and specificity can vary depending on the cell types involved and the specific ND in question. Autophagy, which involves the formation of autophagosomes that degrade damaged organelles and misfolded proteins, is particularly important in neurons due to their post-mitotic nature. This process helps prevent the accumulation of toxic materials that could otherwise lead to neurodegeneration [275]. In contrast, the UPS, which targets proteins for degradation through proteasomes, is essential for the regulation of various cellular processes, including the removal of misfolded proteins and the regulation of signaling pathways critical for neuronal function and survival [276]. In terms of specificity, the functions of autophagy and the UPS can be quite distinct depending on the disease context and the types of cells affected. For example, in AD, autophagy is involved in degrading Aβ plaques, while the UPS regulates tau protein levels and prevents tau aggregation [277,278,279]. Similarly, in PD, both systems manage α-syn aggregates, but their relative contributions can vary based on disease stage and neuronal type [280,281]. Moreover, while autophagy and the UPS sometimes share substrates, such as ubiquitinated proteins and protein aggregates, their roles can be complementary or divergent depending on the disease. For instance, both pathways are engaged in managing protein aggregates in AD and PD, yet they address different aspects of protein quality control and stress responses [279].

In summary, although autophagy and the UPS both play essential roles in neurodegenerative diseases by handling misfolded proteins and damaged organelles, their functions can be specific to different cell types and disease contexts. They share some substrates, particularly those related to protein aggregation, but their mechanisms and impact can vary significantly depending on the type of ND and the specific cellular processes involved [282].

## 7. Conclusions

The involvement of muscle tissue in NDs such as ALS, AD, and PD presents a complex interplay of pathogenic mechanisms that significantly impact patient health and quality of life. Across these conditions, common muscular anomalies such as atrophy, mitochondrial dysfunction, protein aggregation, and inflammation are observed, each contributing to disease progression and symptomatology. However, the underlying molecular pathways and the extent of muscle involvement vary, underlining the importance of disease-specific approaches in understanding and managing these anomalies.

Therapeutic strategies targeting muscle pathology in these diseases show promise in mitigating symptoms and enhancing patient outcomes. Pharmacological interventions aimed at improving mitochondrial function, reducing protein aggregates, and modulating inflammation offer a potential avenue for ameliorating muscle dysfunction. Additionally, physical therapies, including personalized exercise regimens and innovative modalities such as electrical stimulation, have demonstrated beneficial effects in maintaining muscle mass, improving strength, and enhancing overall motor function.

Future research must continue to elucidate the precise mechanisms driving muscle pathology in ALS, AD, and PD to refine these therapeutic approaches. Integrating pharmacological and physical therapies, along with personalized nutritional plans, could provide a comprehensive strategy to address muscle health in NDs. By improving our understanding of muscle involvement and optimizing treatment modalities, we can improve the quality of life for patients and potentially slow the progression of these debilitating conditions.
